# Detection of candidate gene networks involved in resistance to *Sclerotinia sclerotiorum* in soybean

**DOI:** 10.1007/s13353-021-00654-z

**Published:** 2021-09-11

**Authors:** Yu Zhang, Yuexing Wang, Wanying Zhou, Shimao Zheng, Runzhou Ye

**Affiliations:** 1 School of Biological Sciences and Engineering, Shaanxi University of Technology, Hanzhong, 72300 Shaanxi China; 2 College of Agronomy, Xinjiang Agricultural University, Urumqi, 830052 China; 3 Faculté de Médecine Et Des Sciences de La Santé, Université de Sherbrooke, Sherbrooke, QC J1H 5N4 Canada

**Keywords:** *Sclerotinia sclerotiorum*, SNP, Haplotype, Epistasis, Post-GWAS

## Abstract

Quantitative trait locus (QTL) mapping often yields associations with dissimilar loci/genes as a consequence of diverse factors. One trait for which very limited agreement between mapping studies has been observed is resistance to white mold in soybean. To explore whether different approaches applied to a single data set could lead to more consistent results, haplotype-trait association and epistasis interaction effects were explored as a complement to a more conventional marker-trait analysis. At least 10 genomic regions were significantly associated with *Sclerotinia sclerotiorum* resistance in soybean, which have not been previously reported. At a significance level of *α* = 0.05, haplotype-trait association showed that the most prominent signal originated from a haplotype with 4-SNP (single nucleotide polymorphism) on chromosome 17, and single SNP-trait analysis located a nucleotide polymorphism at position rs34387780 on chromosome 3. All of the peak-SNPs (*p*-value < 0.05) of each chromosome also appeared in their respective haplotypes. Samples with extreme phenotypes were singled-out for association studies, 25–30% from each end of the phenotypic spectrum appeared in the present investigation to be the most appropriate sample size. Some key genes were identified by epistasis interaction analysis. By combining information on the nearest positional genes indicated that most loci have not been previously reported. Gene ontology (GO) and Kyoto Encyclopedia of Genes and Genomes (KEGG) enrichment analyses suggest potential candidate genes underlying callose deposition in the cell wall and mitogen-activated protein kinase (MAPK) signaling pathway-plant, as well as plant-pathogen interaction pathway, were activated. Integration of multi-method genome-wide association study (GWAS) revealed novel genomic regions and promising candidate genes in novel regions, which include Glyma.01g048500, Glyma.03g129100, Glyma.17g072200, and the Dishevelled (Dvl) family of proteins on chromosomes 1, 3, 17, and 20, respectively.

**Supplementary Information:**

The online version contains supplementary material available at 10.1007/s13353-021-00654-z.

## Background

White mold (WM) in soybean is a global soybean disease that emerged in the mid-80 s and spread across countries such as the USA, Canada, Argentina, Turkey, Japan, and India (Hoffnan et al. 1998; Ploper 1999). Particularly in North American and Canadian cropping areas, WM has become one of the major factors affecting soybean production. The disease is caused by infection with *Sclerotinia sclerotiorum* (Lib.) de Bary. In 1986, the incidence of this pathogen in Canadian legume production reached 25% (Tu 1986). In recent years, WM has continued to spread, causing an increased harm and serious threats to soybean yield (Koenning and Wrather 2010; Peltier et al. 2012). Taken together, the selecting resistant soybean cultivars is the most economical and effective way to control the disease. Although no soybean line that is completely resistant to *Sclerotinia* has been identified to date, some are known to exhibit partial resistance, a property shown to be a quantitatively inherited trait conferred by multiple genes (Kim et al. 1999; Li et al. 2010; Song et al. 2017). However, the fact that quantitative traits are highly sensitive to environmental factors impedes the accurate selection of target traits under field conditions, thus making it difficult to identify resistant germplasm resources. Therefore, the use of molecular markers to selectively develop disease-resistant cultivars is essential.

Since the first localization of QTL for *Sclerotinia* resistance of soybean by Kim and Diers within the progeny of a biparental cross in 2000 (Kim and Diers 2000), many additional studies have attempted to map loci conferring *Sclerotinia* resistance (Arahana et al. 2001; Li et al. 2010; Guo et al. 2008; Hanet al. 2008; Vuong et al. 2008; Huynh et al. 2010). In the last decade, with the development of next-generation sequencing technologies, genome-wide association studies (GWAS) using SNPs have become the most widely used approach to map traits of interest. Subsequent to the progress made by GWAS in human disease research, this approach has been used in the detection of candidate genes for disease resistance.

Currently, the use of molecular markers for selectively cultivating *Sclerotinia*-resistant soybean has not yet been implemented in practice. Treating the leaves, roots, and stems with oxalic acid and subsequently evaluating the degree of wilting and the level of soluble pigment still represent the main method for selecting soybean cultivars with *Sclerotinia* resistance (Li et al. 2016). In 2010, Li et al. (2011) used SSR markers to discover QTL conferring soluble pigment (SP) content variations that are related to WM resistance and identified linkage groups D1a + q (Gm01), B1 (Gm11), and A2 (Gm08). In a more recent GWAS for WM resistance-associated SP content, 25,179 SNPs from 330 soybean genotypes allowed the detection of a prominent signal on chromosome 13, followed by signals on Gm06, Gm10, and Gm11; 4 candidate genes related to WM disease response and the biosynthetic pathways of anthocyanin were located in the vicinity (< 60 kb) of significant SNPs (Zhao et al. 2015). In 2014, Bastien et al. (2014) used 7864 SNPs of 130 Canadian soybean genotypes by TASSEL3.0 software to elucidate QTL (in order of significance) on Gm15, Gm01, Gm20, and Gm19. In 2015, Iquira et al. (2015) used 8297 SNPs from 101 samples and located QTL (in order of significance) on Gm03, Gm08, and Gm20. Because of differences in soybean type, sample size, phenotype measurement method, marker type and density, and analytical algorithm method, QTL for WM resistance show large variations across studies.

One weakness of working with individual markers/SNPs is that these are not inherited independently; rather, these have a propensity to be inherited in clusters of physically close loci, thus resulting in linkage disequilibrium (LD). Markers that are in high LD within a particular set of materials form haplotypes. The diversity of haplotypes in a genomic region is not limited to the two alleles typically seen for a single SNP locus. As a consequence, haplotypes may more accurately capture the underlying allelic diversity at loci controlling a trait of interest. When used in association tests, haplotypes may therefore better capture the association to the studied trait (Song et al. 2017).

At the same time, most complex traits are determined by the interactions between multiple genes. This is especially true for the phenotypes controlling disease susceptibility, for which associations based on single loci are usually not sufficient to explain or simulate complex traits. Although these alleles are associated with the disease, most of them merely have small effects when considered individually. Gene–gene interactions are often considered to be the cause of the unexplained genetic variations in complex phenotypes, and these interactions are sometimes overlooked in GWAS (Kanishka et al. 2011).

In addition, a challenge in characterizing a complex disease such as WM is the accurate assessment of the degree of resistance. One possible way to facilitate the identification of reliable QTL is to divide the population into two groups expressing extreme phenotypes and discarding those with intermediate phenotypes (Anderson et al., 2010). The use of extreme samples taken from 10 to 35% on either side of the phenotypic distribution curve has previously been reported to be effective for the purpose of identifying associated QTL (Zhang et al. 2011).

To further investigate the QTL conferring partial resistance to WM in soybean, In this study, we employ 20,691 SNPs to reanalysis a previously published dataset that used 7864 SNPs analysis (Bastien et al. 2014), and performed haplotype-trait association analyses, as well as epistatic interaction analysis using both the full genotype set (*n* = 126) and only extreme phenotypes (*n* = 76), which comparing single SNP-trait association tests only by Bastien et al. The detected QTL were compared with the results of other studies, and candidate genes were identified by GO enrichment and KEGG pathway enrichment analyses.

## Materials and methods

### Soybean lines

A panel of 126 soybean lines originate from the article “ genome wide association mapping of sclerotinia sclerotiorum resistance in soybean with a genotyping-by-sequencing approach” (Bastien et al. 2014), in which representative of the diversity present in a private breeding program (Semences Prograin Inc.) in Eastern Canada (see list in Supplementary Table S1).

### Phenotype data

Phenotype data (the stem lesion length) were provided by Bastien(Bastien et al. 2012).

### Genotype data

Single-end sequencing was performed on three lanes of an Illumina HiSeq2000 (at the McGill University-Génome Québec Innovation Center in Montreal, QC, Canada). Data quality control is essential to GWAS. In the present investigation, the *r*^2^ value for linkage disequilibrium, the LOD, and the confidence interval were set to 0.8, 3, and 95%, respectively. HWE (Hardy–Weinberg equilibrium) was not considered. A total of 20,691 SNPs passed the MAF (minor allele frequency) lower limit of 5%; for association, *α* = 0.05 was used as the cut-off significance, which corresponded to a non-adjusted *p*-value of 2.42 × 10^−6^ (0.05/20,691). The 126 samples provided a total of 2,607,066 loci, among which 213,559 (8.192%) were heterozygous.

### Population structure

STRUCTURE 2.3.4 (http://taylor0.biology.ucla.edu/structureHarvesteroybase.org/tools.php) for population structure analysis with the MCMC algorithm and NJ algorithm was used to perform clustering analysis, and phylogenetic reconstruction was conducted using MEGA5.05 (http://www.megasoftware.net/). SPSS was used for principal component analysis (https://www.ibm.com/analytics/us/en/technology/spss/). The Bayesian clustering algorithm implemented in STRUCTURE 2.3.4 was used to simulate population genetic structure. According to the *Q* value distribution of each population, it is considered that the sample has a single blood relationship when the *Q* value of a sample in a population is greater than or equal to 0.6, and it is considered that the sample has mixed sources when *Q* value is less than 0.6. To obtain an estimate of the most probable number of population (*K*), *K* values from 1 to 10 were simulated with 20 iterations for each *K*, using 10,000 burn-in periods followed by 10,000 Markov Chain Monte Carlo iterations. Delta *K* was plotted against *K* values and the best number of clusters was determined following the method proposed by Evanno et al. (2005), and population structure diagram was obtained by the Structure Harvester platform (Earl and Vonholdt 2012).

### Association analysis

For complete phenotype set, single SNP-trait association tests, with additive effects only and additive effects plus dominant effects of GLM (general linear model) or MLM (mixed linear model) model, were run in TASSEL v.5.0 (http://www.maizegenetics.net/tassel) in max(*T*) permutation mode using a permutation test with 10,0000 times, and for haplotype analysis using PLINKv1.07 (http://www.softpedia.com/get/Science-CAD/PLINK.shtml), the command line “–blocks” was used to assign SNPs to their respective haplotypes. Then, analysis for haplotype-trait association was performed, and haplotype-trait association analysis only for additive effects.

The frequency distribution of the genotypic segregation in the two groups (high-value group and low-value group, namely extreme phenotypes that were distributed at both ends of 126 phenotypic spectrum, it can be seen as reduced data set, we treat it as a quality character, assuming a disease phenotype as 1 = unaffected, 2 = affected, 0 = miss) will thus deviate from the Mendelian law. Using a chi-square test to assess this deviation for either or both groups, it was then possible to infer whether the marker is linked to the QTL. In the analysis with extreme phenotypes as implemented in HAPLOVIEW4.2 (http://www.broadinstitute.org/haploview), because sample size was reduced without a decrease in the number of markers used, an increase in the number of false positives resulted from higher inflation; *p*-values were thus adjusted through a 10,000-time permutation test approach. With the 95% CI of *D*’ value bound between 0.70 and 0.98 for adjacent SNPs to infer haplotype blocks, then carry out haplotype-phenotype association tests of extreme phenotype set.

### SNP × SNP epistatic interaction analysis

PLINK v1.07 has different modes for specifying which SNPs are tested. To increase the power of epistasis detection, three analytical methods were used: (1) all SNP-by-all SNP two-locus epistasis test between all of the 205,284,045 SNP pairs; (2) SNP-by-SNP epistasis test between only significant SNPs pre-filtered by genome-wide association analyses; and (3) SNP-by-all epistasis test between significant SNPs and all SNPs. CYTOSCAPE v3.7.1 was used to visualize the interaction network.

## Results

### LD decay and haplotype construction

**Fig. 1:**
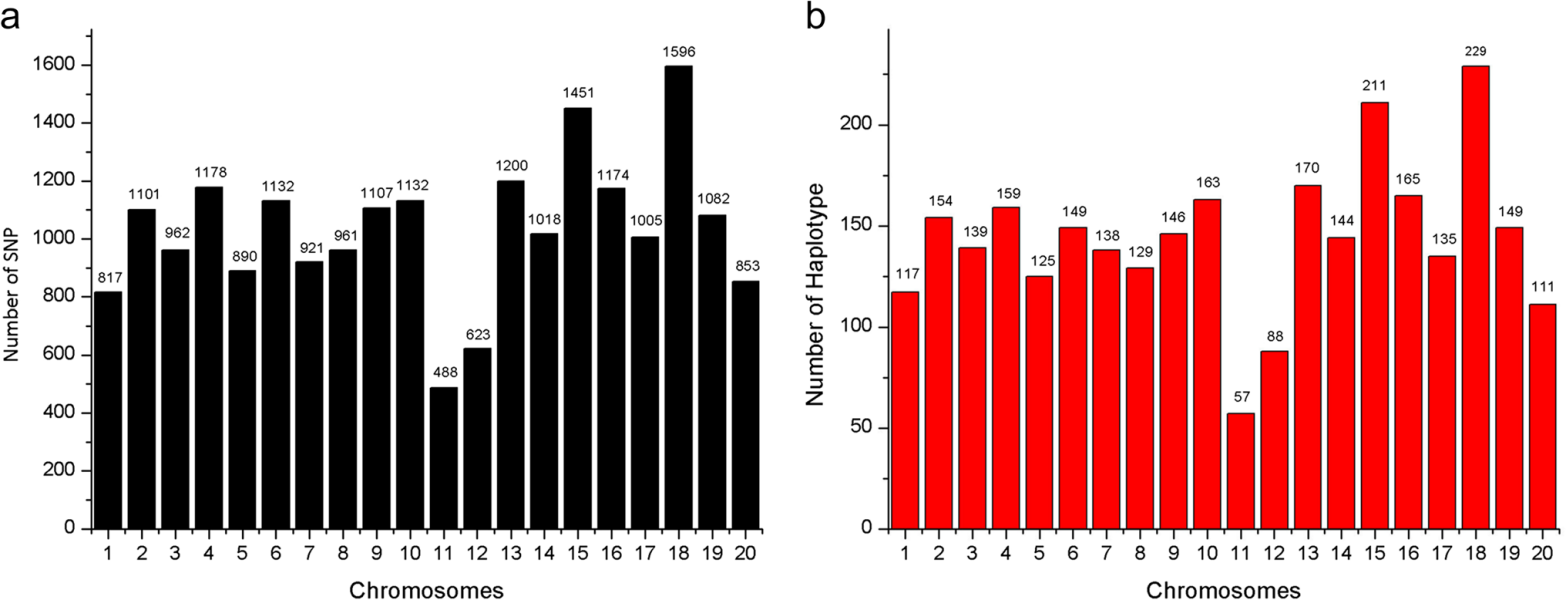
Distribution of SNP/haplotype: (**a**) Number of SNP on each chromosome. (**b**) Number of haplotype on each chromosome

To compare the results of QTL mapping analyses conducted either using individual SNP markers or haplotypes, we first needed to assemble a catalogue of haplotypes for our association panel comprising 126 soybean lines. Genotyping-by-sequencing was used to obtain SNP data for this panel of soybean lines and these were then used to construct haplotypes based on the observed LD. At *r*^2^ > 0.2, 84,255 pairs of SNPs were found to be in LD. Among the 84,255 marker pairs in LD, an average *r*^2^ of 0.73 was observed; 46,322 pairs (55.0%) had *r*^2^ ≥0.8, and 7841 pairs (9.3%) showed an *r*^2^ value of 1. The distribution of LD along each chromosome was uneven. The average physical distance between pairs of loci decreased as their LD increased. Pairs with *r*^2^ < 0.8 were 154.1 kb apart, pairs with *r*^2^ ≥ 0.8 were 139.7 kb apart, and pairs with *r*^2^ = 1 were separated by 99.6 kb. The 20 chromosomes yielded a total of 2,858 predicted haplotypes (Fig. 1), with chromosome 18 possessing the most haplotypes (229) and chromosome 11 possessing the least (57). The largest haplotype was composed of 36 SNPs, whereas the smallest haplotype comprised only 2 SNPs, and a haplotype consists of 5 SNPs on average. The average length of the haplotype is 64.4 kb; the longest haplotype spanned 200.0 kb, whereas the shortest encompassed only 2 bp. The distribution of SNPs along each chromosome was uneven, where chromosome 18 contained the largest number of markers (1596), whereas chromosome 11 included the fewest (488).

## Population structure analysis

**Fig. 2:**
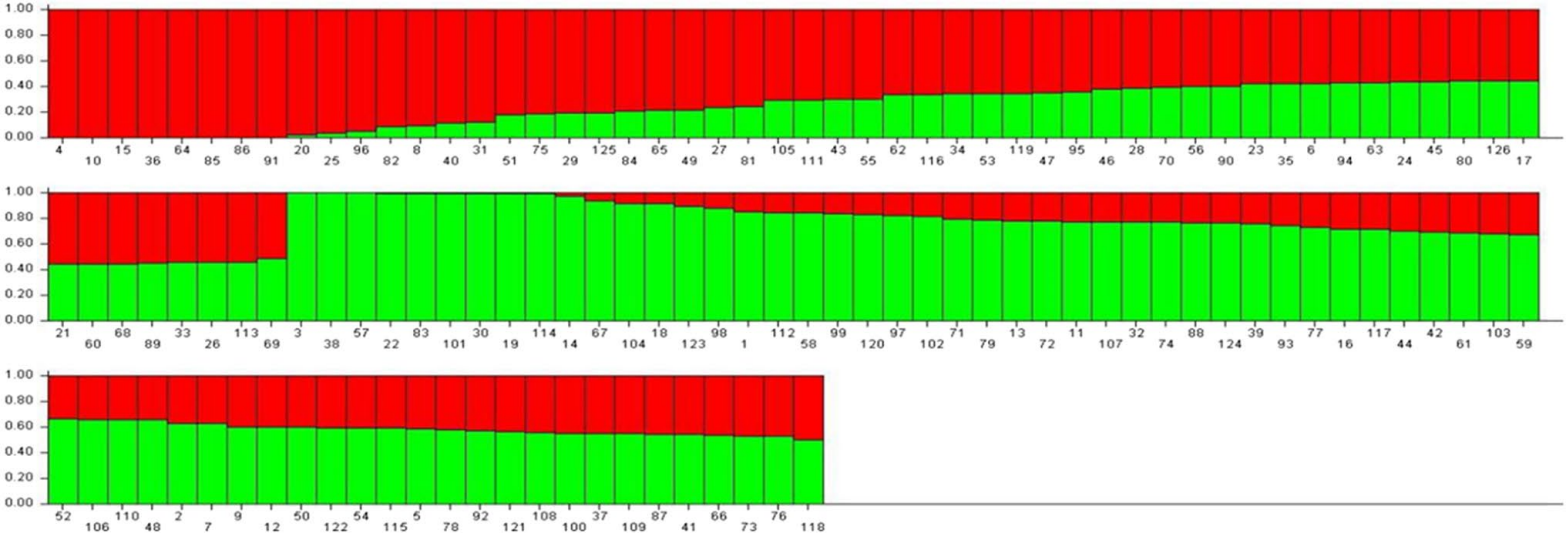
Population structure diagram of the 126 soybean lines. Note: Red: group I; Green: group II. Vertical lines on the *X*-axis refer to each variety. The proportion of each color represents probability rate with which a given genotype belongs to each group

Population genetic structure was performed based on the Bayesian method. Delta *K* reached a maximum value at *K* = 2, suggesting that the 126 soybean lines were divided into two subgroups (consisting of 58 and 68 samples) (Fig. 2).

In addition, the contribution rates of the first three PC (principal component) were 6.24%, 5.44%, and 5.16%, respectively, and the 126 lines cannot be clearly grouped by PC method, while the 126 soybean genotypes into two groups using NJ (neighbor joining). However, the genetic distance between these two groups was not significant, indicating that the samples have a low level of genetic stratification.

### Genome-wide association study approaches

The results showed that the resistance to *Sclerotinia* infection among the 126 soybean lines was significantly different, in which varied from 28.6 to 192.4 mm, with the traits of continuous distribution and quantitative inheritance.

### Complete phenotype set association analysis

#### Single SNP-trait association analysis

Six different modes were tested: (1) GLM; (2) GLM (PC); (3) GLM (*Q*); (4) MLM (*K*); (5) MLM (PCA: principal component analysis + *K*); and (6) MLM (*Q* + *K*). In this study, the number of principal components is selected according to *Q*-*Q* Plot (quantile–quantile plots) (Figure S1), and *Q*-*Q* Plot revealed that the model using the first 79 PCs, which altogether captured 85% of the population structure, produced results that correlated most closely with the expected *p*-values (TableS2, S3). Thus, the first 79 PCs were used to capture population structure in the association analyses. To demonstrate the reliability of our results, significance level was evaluated by Bonferroni correction and permutation tests with 10,0000 permutations. The strongest signal came from the SNP at position rs34387780 on chromosome 3 by GLM, followed by those from markers on chromosomes 20, 1, 4, and 17 (Table 1).

**Table 1:** Single SNP-trait association of 126 lines using additive effects and additive effects + dominant effects (*P*-value < 0.05)

GLM (general linear model)							
Additive effects only				Additive effects + dominant effects			
Chromosome	Position	*P*-value (permutation)	*P*-value (Bonferroni-adjusted)	Chromosome	Position	*P*-value (permutation)	*P*-value (Bonferroni-adjusted)
3	34,387,780	0.00816	0.01788	20	42,100,739	0.0132	0.0329
3	34,387,823	0.0086	0.01788	20	42,091,969	0.01418	0.0356
3	34,387,841	0.00816	0.01788	20	42,118,002	0.02327	0.059
20	42,091,969	0.01211	0.02673	18	55,469,724	0.03058	
1	5,589,867	0.0124	0.02752	1	5,589,867	0.03392	
20	42,118,002	0.01641	0.03613	3	34,387,780	0.04016	
3	34,395,745	0.01695	0.0376	3	34,387,823	0.04016	
3	34,387,945	0.0243		3	34,387,841	0.04016	
20	42,100,739	0.03358		3			
20	42,122,908	0.03878		3			

**Fig. 3:**
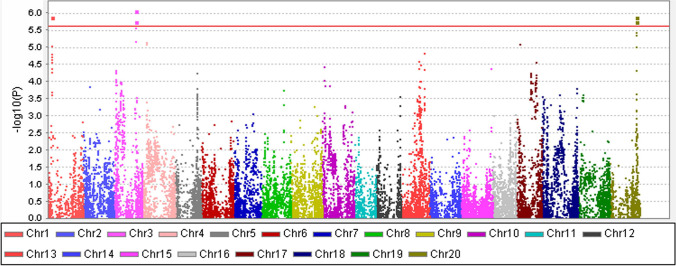
Manhattan plot of the association between single SNP-Trait. Note: *P*-values for the association of each single nucleotide polymorphism with phenotype are shown on the *y*-axis. The SNPs are plotted on the *x*-axis according to their chromosomal location. Blue line: *α* = 0.05; red line: *α* = 0.01 (additive effects only)

The loci showing the most significant association signals included one SNP located at position rs34387780 on chromosome 3 and five other markers in close vicinity (Fig. 3).

#### Haplotype-trait association analysis

For haplotype-trait association analysis, one signal from chromosome 17 represented the most significant association and accounted for 17.56% of the phenotypic variations. Less significant haplotypes were also found on chromosome 1 (Table 2).

**Table 2:** Haplotype-trait analysis of 126 lines (*p*-value < 0.05)

Chromosome	Position of haplotype	Haplotype (1 = A, 2 = C, 3 = G, 4 = T)	*R*^2^	*P*-value (Bonferroni-adjusted)
17	5,575,883|5,647,814|5,648,648|5,734,897	3114	0.1756	0.021
1	5,589,867|5,700,523|5,724,122|5,724,140	1223	0.1648	0.049

### Extreme phenotypes set association analysis

#### Single SNP-trait association analysis

The present investigation attempted using extreme phenotype groups for association analysis. We utilized 10%, 20%, and 30% of the 126 soybean lines as cut-off thresholds for defining membership to the two extreme sample groups. Results from association analyses showed that all significant signals detected using both the 10%- and 20%-extreme groups were represented in those identified using the 30%-extreme groups; hence, only the latter was used for further analyses. The strongest association was found on chromosome 1, followed by those on chromosomes 20, 10, 17, 13, and 4 (Table S4).

#### Haplotype-trait association analysis

The strongest association was identified for a haplotype formed by 12 SNPs on chromosome 1; haplotypes on chromosomes 13, 4, 10, 20, and 17 were also detected as having significant association to soybean lesion length (Table S5).

The analyses using extreme phenotype groups (both single SNP-trait and haplotype-trait) revealed that the peak-SNPs in the single SNP-trait analysis also appeared in their respective haplotypes. Because the method using extreme groups suffered from a diminished number of soybean genotypes, the analysis using haplotypes had proven to be advantageous by reducing the degree of freedom; hence, the adjusted *p*-value threshold for the haplotype-based analysis was lowered to *α* = 0.01 for subsequent candidate gene selection.

**Fig. 4:**
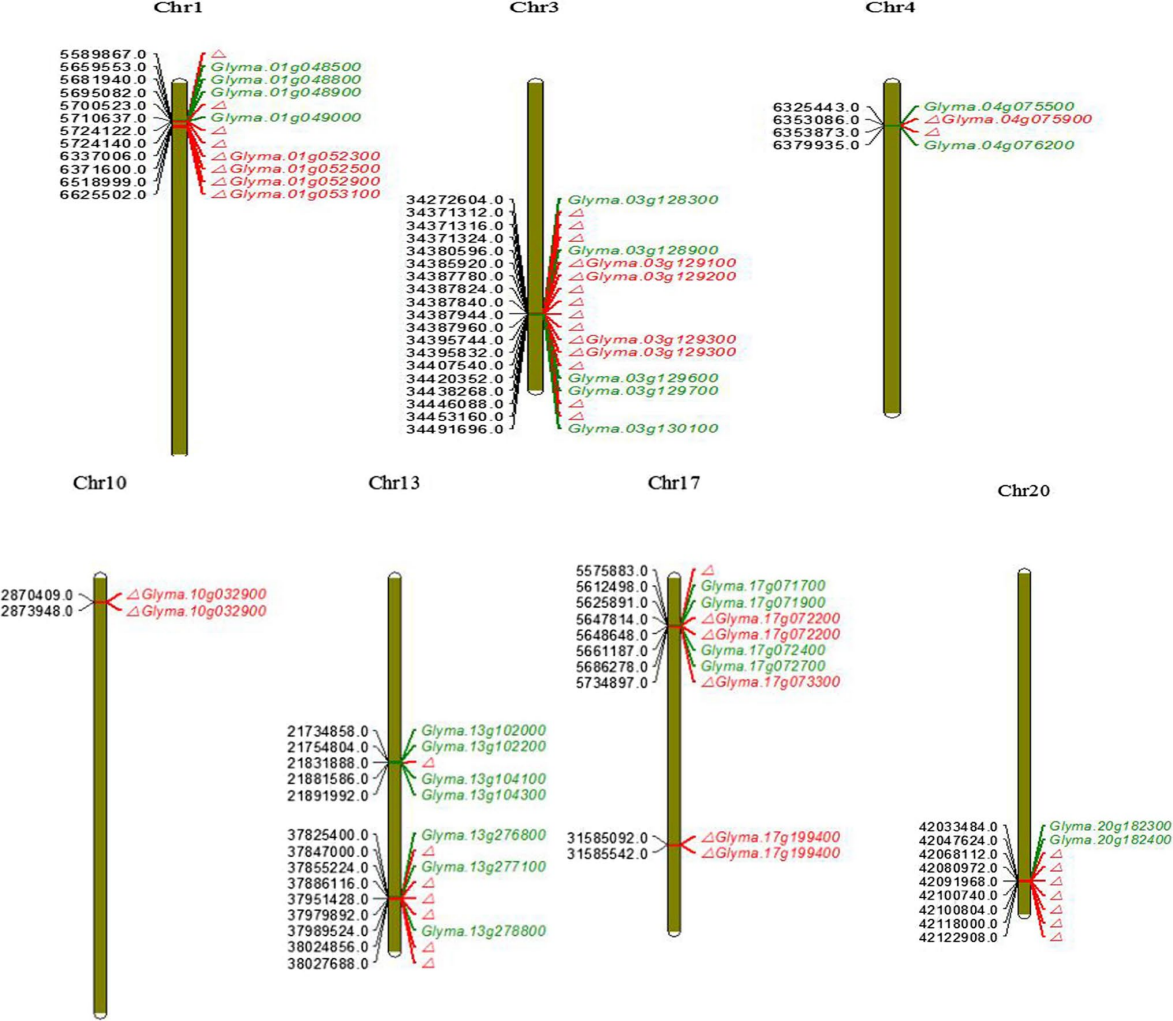
Prioritizing candidate genes. Note: Triangle represents the position of haplotype, red font indicates QTL is within a gene, and green font indicates that the QTL is outside a gene

The results of QTL mapping for *S.* *sclerotiorum* resistance in soybean using the abovementioned methods are shown in Fig. 4.

### SNP × SNP epistatic interaction analysis

**Fig. 5:**
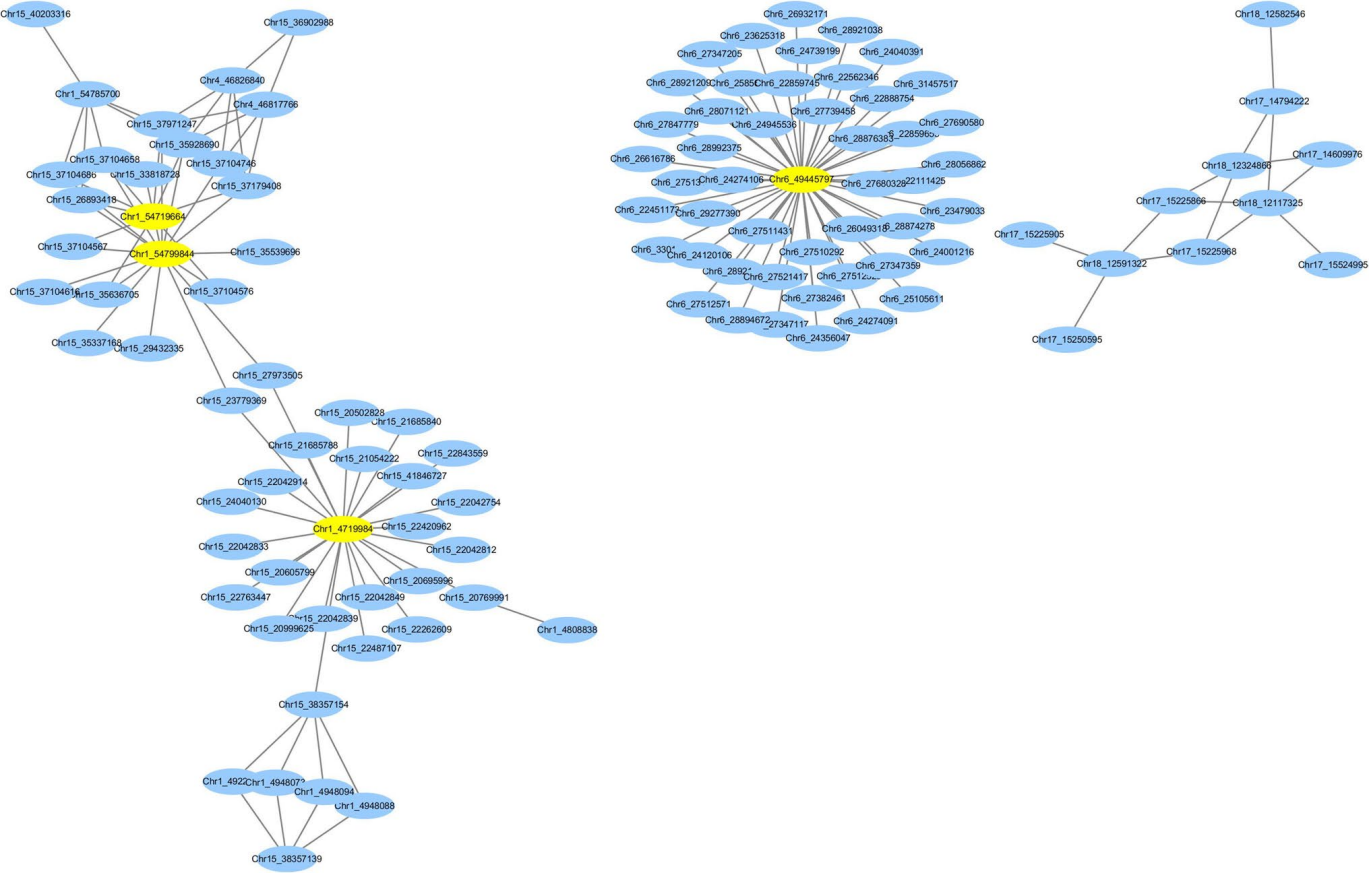
The top three epistasis interaction subnetworks

With methods (2) and (3), the use of different *p*-value thresholds for declaring significant association and for defining significant SNP groups yielded different results. With a single-SNP cut-off *p*-value of 1 × 10^−4^, neither method (2) nor method (3) produced significant epistatic interactions. Thus, we used the ALL × ALL epistasis mode by PLINK v1.07 and performed a total of 205,284,045 valid SNP × SNP tests. A total of 220 SNPs were involved in the interaction network (cutoff *p*-value < 0.07) (Table S5), with 112 SNPs in the first three subnetworks. Multiple interactions were found between Gm01 and Gm15 and within Gm06 (Fig. 5). In the first sub-network, there were 9, 17, and 24 proteins interacting with SNP Chr1-rs54719664, Chr1-rs54799844, and Chr1-rs4719984 based on shortest path search, respectively, of which high-node genes in the network may regulate other genes or be regulated by other genes. Among these, Chr1-rs54719664 is closely linked to the *Glma.01g216800* and *Glma.01g216900* gene, which were annotated as cytochrome P450, family 87, subfamily A, and polypeptide 2. Chr1-rs54799844, a SNP locus within the *Glma.01g217900* gene, was annotated as transcription initiation factor TFIIE, beta subunit. Chr1-rs4719984 is closely linked to *Glma.01g043300* and *Glma.01g043400* genes, which were annotated as WRKY DNA-binding protein 72 and cystathionine beta-synthase (CBS) protein, respectively.

For further analysis, the asymptotic *p*-values produced by the all SNP-by-all SNP interaction analysis that were smaller than 1 × 10^−8^ were considered (Table 3).

**Table 3:** Top genes in the epistasis network (*P* < 1 × 10^−8^)

ALL × ALL										
SNP1					SNP2					
CHR1	Position	Distance to SNP (bp)	Candidate gene	Annotation	CHR2	Position	Distance to SNP (bp)	Candidate gene	Annotation	*P*-value
6	563,504	Within	Glyma.06g007400	Vacuolar-type H( +)-ATPase C3	18	57,104,652	2087	Glyma.18g292800	S-adenosyl-L-methionine-dependent methyltransferases superfamily protein	2.475E − 9
7	41,611,205	3442	Glyma.07g235100	Molybdopterin biosynthesis MoaE family protein	8	8,785,343	12,370	Glyma.08g114700	Calmodulin-binding family protein	2.652E − 9
1	54,799,844	2853	Glyma.01g219000	Transcription initiation factor TFIIE, beta subunit	15	26,893,418	60,406	Glyma.15g204300	Surfeit locus protein 5 subunit 22 of mediator complex	2.827E − 9
6	563,504	29,305		Arginine decarboxylase 2	18	57,114,367	11,802		S-adenosyl-L-methionine-dependent methyltransferases superfamily protein	5.596E − 9
1	54,799,844	2853		Transcription initiation factor TFIIE, beta subunit	15	37,971,247	49,861		Ca(2)-dependent phospholipid-binding protein (Copine) family	6.06E − 9
1	54,719,664	1102		Cytochrome P450, family 87, subfamily A, polypeptide 2	15	26,893,418	60,406		Surfeit locus protein 5 subunit 22 of mediator complex	6.536E − 9

### Gene assignment and functional annotations

We integrated the results of the commonly detected loci/regions, including genes by epistasis interaction analysis (*P* < 10^−7^) and the candidate genes potentially involved in the defense mechanism of soybean to *Sclerotinia* infection (Table 4).

**Table 4:** Prioritizing candidate genes(*P* < 1 × 10^−7^)

Chr	Position of haplotype	Candidate gene	Annotation
3	34,371,310/34371316/34371324/34385919/34387780/34387823/34387841/34387945/34387962/34395745/34395833/34407540/34446090/34453159	1.Glyma.03g128300 2.Glyma.03g128900 3.Glyma.03g129100 4.Glyma.03g129200 5.Glyma.03g129300 6.Glyma.03g129600 7.Glyma.03g129700 8.Glyma.03g130100	1. Glutamate synthase 2. Lycopene cyclase/lycopene β-cyclase 3. Pyrroline-5- carboxylate (P5C) reductase 4. Cytochrome P450, family 86, subfamily A, polypeptide 1 5. S-adenosyl-L-methionine-dependent methyltransferases superfamily protein 6. Glutaredoxin family protein 7. Cystathionine beta-lyase (CBL) 8. Calcium-dependent phosphotriesterase superfamily protein
17 (01) 17 (02)	5,575,883/5647814/5648648/5734897 31,585,092–31,585,542	1. Glyma.17g071700 2. Glyma.17g071900 3. Glyma.17g072200 4. Glyma.17g072400 5. Glyma.17g072700 6. Glyma.17g073300 7. Glyma.17g199400	1. Leucine-rich receptor-like protein kinase family protein 2. Calcium-binding EF-hand family protein 3. Cellulose synthase family protein 4. Heat shock protein 70B 5. Nucleic acid binding 6. Signal recognition particle receptor alpha subunit family protein 7. Glutathione S-transferase THETA 2
20	42,068,110/42080972/42091969/42100739/42100805/42118002/42122908	1. Glyma.20g182300 2. Glyma.20g182400	1. DVL family protein 2. Ras-related small GTP-binding family protein
1 (01) 1 (02)	5,589,867/5700523/5724122/5724140 6,337,006–6,625,502	1. Glyma.01g048500 2. Glyma.01g048800/Glyma.01g048900 3. Glyma.01g049000 4. Glyma.01g052300 5. Glyma.01g052500 6. Glyma.01g052900 7. Glyma.01g053100	1. Galactosyltransferase1 2. Glucose-methanol-choline (GMC) oxidoreductase family protein 3. Glutathione S-transferase THETA 3 4. Levansucrase 5. GDP-fucose protein O-fucosyltransferase 6. Early-responsive to dehydration stress protein 7. Argonaute family protein
4	6,353,086/6353873	1. Glyma.04g075500 2. Glyma.04g075900 3. Glyma.04g076200	1. Plant calmodulin-binding protein-related 2. Peptidyl-tRNA hydrolase II (PTH2) family protein 3. WRKY DNA-binding protein 11
13 (01) 13 (02)	21,831,889 37,847,000/37886116|37,951,427|37,979,893|38,024,854|38,027,686	1. Glyma.13g102000 2. Glyma.13g102200 3. Glyma.13g104100 4. Glyma.13g104300 5. Glyma.13g276800 6. Glyma.13g277100 7. Glyma.13g278800	1. WRKY DNA-binding protein 11 2. Protein kinase superfamily protein 3. Polyamine oxidase 5 4. Protein kinase superfamily protein 5. Protein kinase superfamily protein 6. Cytochrome P450, family 72, subfamily A, polypeptide 15 7. Leucine-rich repeat (LRR) family protein
10	2,870,409–2,873,948	Glyma.10g032900	WRKY DNA-binding protein 21

**Fig. 6:**
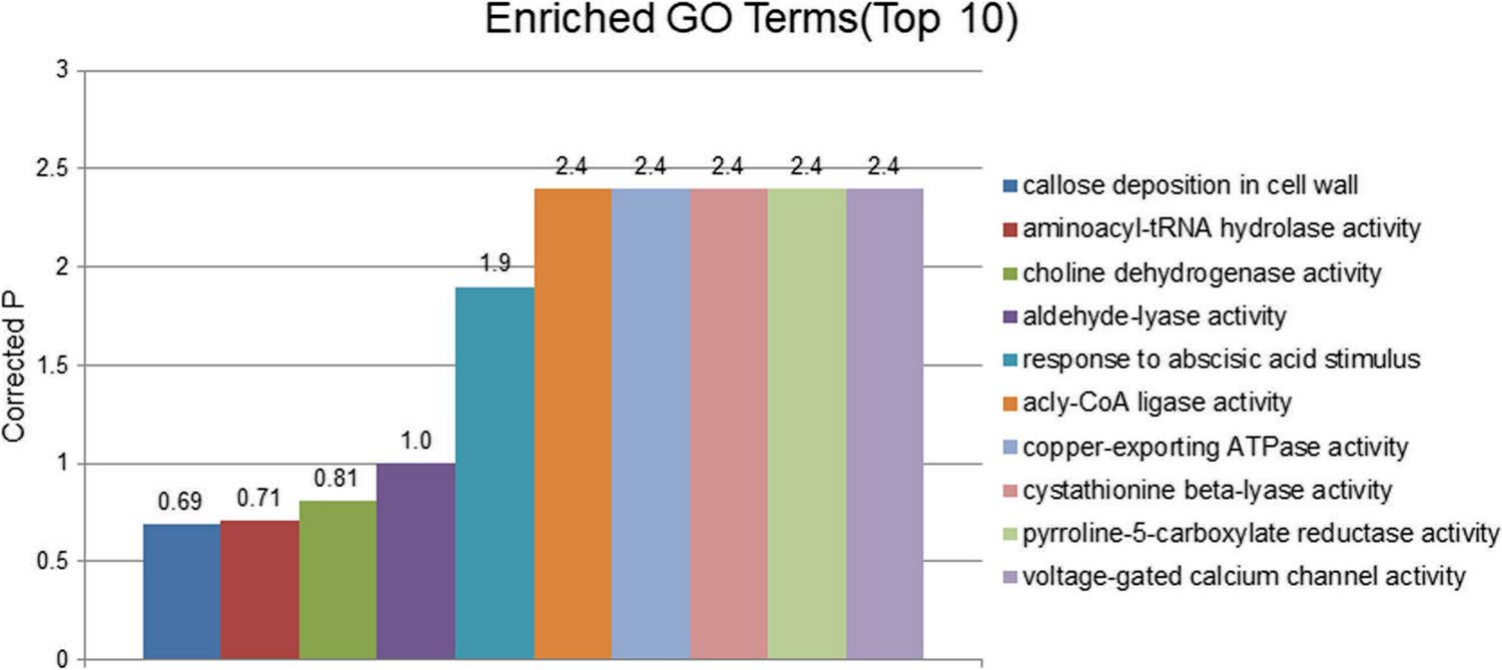
The results of GO functional enrichment analysis

**Fig. 7:**
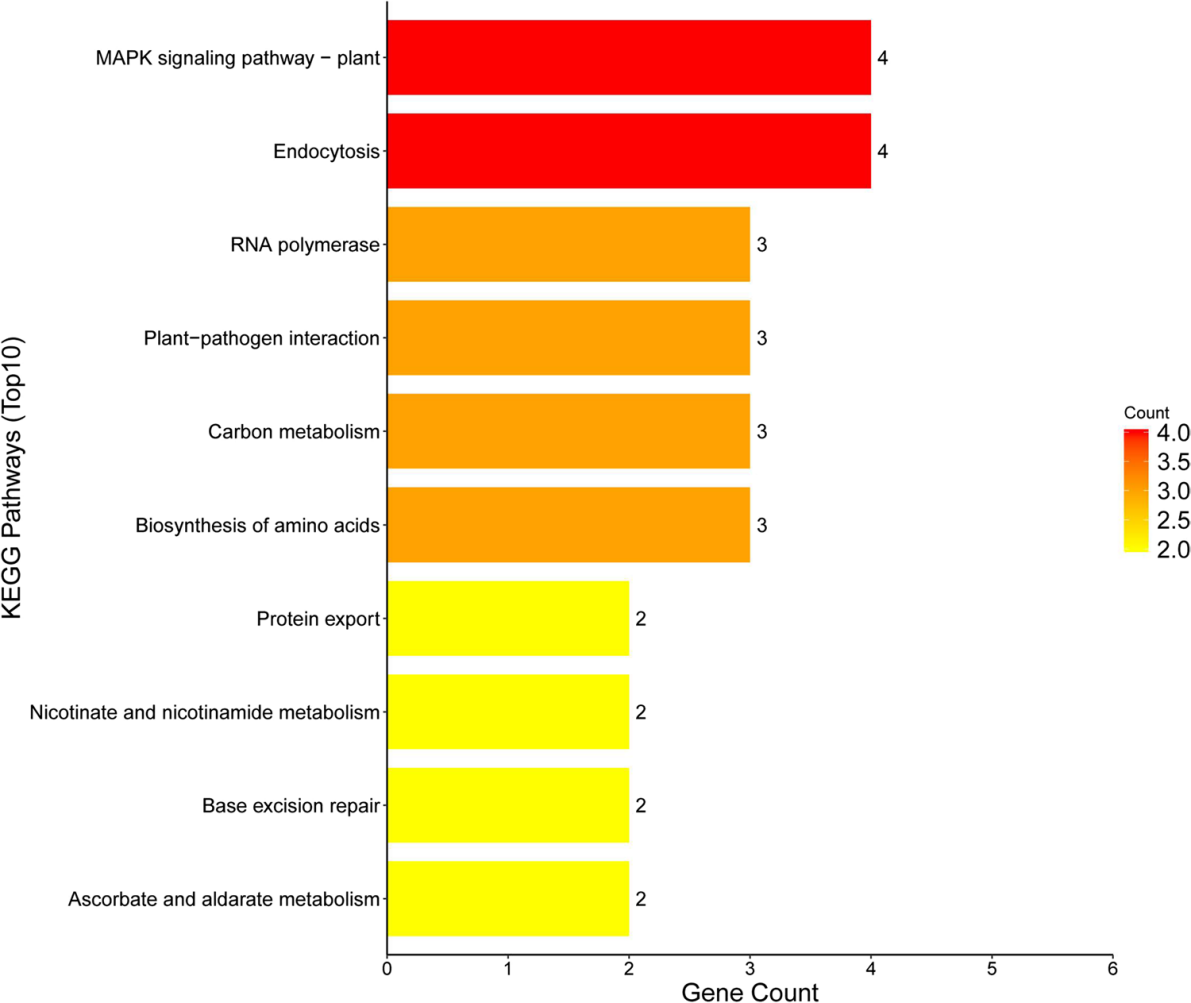
The results of KEGG pathways enrichment analysis

These can be classified into genes encoding signal transduction molecules, transcriptional regulators, disease resistance proteins, and proteins of unknown functions. These positional candidate genes were further prioritized using GO enrichment analysis by the SoyBase Analysis Tools (http://soybase.org/tools.php) (Fig. 6) and KEGG web platform (https://www.genome.jp/kegg/tool/map_pathway2.html) (Fig. 7), including 241 genes in GO annotation, which resulted in different significantly enriched GO terms and KEGG pathways relevant to *S.* *sclerotiorum*-related mechanisms. Among the enriched GO terms and pathways were callose deposition in cell wall-related such as “GO:0,052,543”; signal transduction-related such as “GO:0,009,737 ~ response to abscisic acid stimulus” and “GO:0,005,245 ~ voltage-gated calcium channel activity”; biosynthetic process-related such as “GO:0,006,537 ~ glutamate synthase” and “GO:0,009,833 ~ Cellulose synthase family protein”; and response to stress-

related such as GO:0,004,735 ~ pyrroline-5-carboxylate reductase.”

## Discussion

The phenotypic data identification is very important in genome-wide association studies, because the determination of the phenotypic data of 126 soybean lines used in this study had previously been performed in a greenhouse to ensure equal selective pressure, environmental variability that affects phenotypic variation can be ignored, and that ensure the reliability of phenotypic value, those disease resistance lines such as Karlo RR, PRO 275, and Toma, PS73, Nattosan, and Supra as susceptible ones; however, Williams 82 was in the middle phenotypic value on stem lesion lengths in response to *Sclerotinia* infection.

Quantitative traits are controlled by many genes that participate in multiple signaling pathways. QTL analysis based on genome-wide essentially consists of a statistical measure that is based on probabilistic criteria and identifies a set of genomic segments that may influence a given quantitative trait. In theory, the reliability of the analysis is thus positively correlated with both sample size and the number of polymorphic loci. The associated regions revealed herein are more abundant and different from those identified in previous investigations. Although we analyzed the same soybean samples used by Bastien et al. (2014), differences in analytical method and marker density have marked repercussions on the outcome of GWAS. In terms of marker density, the mean inter-SNP distance was 43.3 kb in the present study, whereas Bastien et al. reported 238.4 kb. Hence, this 5.5-fold increase in marker density leads to the discovery of association loci/regions that have not been previously identified by Bastien et al. Among the 4 QTL detected by Bastien et al. [Gm15:13,651,235 (V1.0)–13,666,875 (V2.0), Gm01:29,185,984 (V1.0)–27,657,519 (V2.0), Gm20:39,698,515 (V1.0)–40,820,870 (V2.0), and Gm19:50,557,054 (V1.0) – 50,677,474 (V2.0)], only the SNPs on chromosomes 15 and 1 appeared in the QTL mapping results of this study, but these showed low association to WM (Gm15: *P*_unadjusted_-value = 0.06; Gm1: *P*_unadjusted_-value = 0.5189). The other two SNPs (on chromosomes 20 and 19), similar to the four QTL identified by Iquira et al. (2015), were not included in the results of this study. The present study used stem lesion length after inoculation with *S.* *sclerotiorum* as phenotype, whereas Li et al. (2016) and Zhao et al. (2015) based their association analysis on soluble pigment content in soybean stems because sample type, sample size, method of obtaining phenotype, marker density, and analytical method were dissimilar, and different QTL maps were also generated.

Furthermore, previous studies have only analyzed the association between single-locus and trait, whereas the present study also investigated the genome-wide distribution of haplotypes in soybean and their association with lesion length due to *Sclerotinia* infection. All of the peak-SNPs appeared in the corresponding haplotypes; the 6 SNPs from positions rs34387780 to rs34395745 formed a haplotype on chromosome 3. Because some of these SNPs are heterozygous in some materials, we observed slight differences in the *p*-values obtained using single-SNP and the corresponding haplotype. Some studies were conducted for genomic regions spanning 100–300 kb around single association loci (Zhao et al. 2015), effectively introducing the concept of haplotypes. However, the association of haplotype-trait may increase the detection rate of true disease-related QTL because haplotypes encompass the genetic information of multiple SNPs. It can be seen from the genes identified through the haplotype-based association that the disease-related genes tend to form a gene cluster, which acts synergistically in disease response. In addition, the analysis of the haplotype-phenotype association improves our understanding of this disease response pathway, in which haplotypes are likely to work as functional units. In addition, by being able to reduce the degree of freedom, haplotypes yielded better statistical and analytical effects than single-SNPs.

The strongest epistasis interaction was observed between the vacuolar-type H ( +)-ATPase C (V-H + -ATPase C) gene on chromosome 6 and the S-adenosyl-L-methionine-dependent methyltransferases superfamily protein (SAM-Mtases) gene on chromosome 18. The V-H + -ATPase C subunit is the most sensitive subunit in the plant’s response to stress. During periods of stress, the number of C subunits and their mRNA levels drastically and rapidly increase (Xu et al. 2011). The S-adenosyl-L-methionine–dependent methyltransferases superfamily protein (SAM-Mtases) gene has direct effects on the synthesis of many secondary metabolites, such as lignin and flavonoids, and it plays an important role in plant physiological processes involved in hormonal growth and insecticidal, antibacterial, and disease resistance behaviors. Furthermore, arginine decarboxylase, on chromosome 6, is a key enzyme in polyamine biosynthesis. The epistatic interaction between the two genes on chromosomes 6 and 18, coupled with the polyamine oxidase gene identified on chromosome 13, suggests that the polyamine biosynthetic pathway participates in the resistance of soybean to *Sclerotinia* infection (Scandiani et al. 2015).

Molybdenum is one of the essential trace elements in plants; its physiological functions of molybdenum in plants are mainly achieved through molybdenum-containing enzymes. Studies in wheat indicate that molybdenum is associated with abiotic stress and activity of resistant enzymes (Al-Issawia et al. 2016). The interaction analysis on chromosomes 7 and 8 suggests that molybdenum cofactors, which can be combined with many molybdenum enzymes that induce physiological and anti-stress functions, play a role in the infection of soybean by *Sclerotinia* through Ca^2+^-signal transduction.

Cytochrome P450 is involved in plant responses to abiotic and biological stressors (Jarsch and Ott 2011; An et al. 2017). Remorin is a family of N-terminal proline-rich membrane proteins; in particular, the proline content of group 1b remorin protein in dicotyledonous plants is twice that of group 1a remorin in other organisms (Raffaele et al. 2007). Additionally, the identification of pyrroline-5-carboxylate reductase (*P5CR*), the enzyme that catalyzes the final step of proline synthesis through single-SNP association analysis, is also corroborated by the fact that remorin is a proline-rich protein. The interaction between plant remorin protein and bacteria has already been investigated (Lefebvre et al. 2010; Tóth et al. 2012). Using maize as a model, Jamann et al. (2016) were the first to confirm that an interaction exists between remorin and fungus. Previous studies have shown that members of the copine family may play a role in Ca^2 +^ signaling; thus, given the interaction detected between the genes on chromosomes 1 and 15, these genes may be implicated in the response of soybean to *Sclerotinia* infection.

Therefore, it is very important to perform interaction analysis between SNP pairs. With epistatic interaction analysis, some genes that respond to plant abiotic stress and participate in anti-stress defense responses were uncovered. A relatively large body of evidence supports the involvement of P450 expression in host–pathogen interactions (Motallebi et al. 2015). None of the identified single-SNP association loci appeared in loci that were detected by epistatic interaction analysis; this is probably because only two-locus epistasis test models of association were used, whereas the complex phenotype of *Sclerotinia* resistance results from the interaction of multiple loci.

Enrichment analysis indicated that calcium-binding protein, leucine-rich repeat receptor-like protein kinase, protein kinase superfamily protein, small GTP-binding protein, transcription factors (WRKY, zinc finger protein, heat shock transcription factor), and glycosyltransferases (levansucrase and GDP-fucose protein O-fucosyltransferase) are involved in plant signal transduction. Cellulase synthase, glutamate synthase, P5CR, lycopene β-cyclase, polyamine oxidase, SAM-Mtases, glucose methylcholine oxidoreductase family protein, glutathione-S-transferase, cytochrome P450, NBS-LRR disease resistance protein, and Argonaute family protein probably participate in the disease resistance mechanisms of soybean to *Sclerotinia* infection. Among these, calcium-binding protein, SAM-Mtases, cytochrome P450, WRKY, glutathione-S-transferase, leucine-rich repeat receptor-like protein kinase, glycosyltransferase, and protein kinase superfamily protein were detected multiple times in the association analysis. The Ca^2 +^ signaling pathway is a very important transduction pathway that plays an important regulatory role in soybean’s resistance to *S.* *sclerotiorum* (Zhou et al. 2013); this is corroborated by the repeated detection of CaM and CDPK. The effect of SAM-Mtases on disease resistance may be mediated by the synthesis of plant lignin and their participation in the regulation of flavonoid metabolism; the SAM-Mtases gene is hence expected to be one of the important candidate genes for plant disease resistance. P450s have been isolated from host–pathogen interactions, some of which are as toxins and some as stress signals that activate anti-disease responses. WRKY transcription factors form a large family of plant transcription factors. Studies have shown that WRKY proteins are a key component of plant stress response. In plants, the WRKY transcription factor is most abundant in soybean. WRKY belongs to a broad class of transcriptional regulators, plays an important role in a variety of signal transduction pathways, and has been reported in various studies on plant responses to adverse conditions (Wen et al. 2017). The expression of WRKY6, WRKY8, and WRKY11 was significantly upregulated in *Arabidopsis* after inoculation with *S.* *sclerotiorum*; the WRKY gene may thus achieve its anti-*Sclerotinia* control function by activating multiple pathways (Zhao 2012.). The role of the Ca^2+^ signaling pathway and the WRKY11 gene in the plant’s anti-Sclerotinia property has been demonstrated (Wang 2015). Glutathione S-transferase (GST) on chromosomes 17 and 1 may be extensively involved in the lipid peroxide decomposition during oxidative-stress caused by soybean’s response to *Sclerotinia* infection (Ferreira et al. 2010). Through modification of plant hormones and hypersensitivity responses, glycosyltransferase can antagonize essential pathogenic factors to maximize disease resistance (Meng et al. 2013). Consequently, the three glycosyltransferases on chromosome 1, galactosyltransferase, levansucrase, and GDP-fucose protein O-fucosyltransferase may affect the resistance of soybean to *Sclerotinia* by various glycosylation reactions.

Plants do not have a strong immune system; thus, their own defense mechanism is hence particularly important, with the cell wall is the first barrier of plant against pathogen infection Studies have confirmed that epidermal and cortical cells of resistant varieties make use of cytoplasmic disintegration and enhanced cell walls to delay pathogen impregnation and to increase pathogenic resistance (Valera 2014); this is consistent with the results of GO analysis in this study. During pathogenesis, *Sclerotinia* induces cellulose fiber degradation to soluble disaccharide through secretion of cellolase, and then degradation of cellobiose to glucose by β-glucosidase. Experiments show that the pathogenicity of the *S.* *sclerotiorum* strain in rape is positively correlated with its cellulase activity (Mao et al. 2011). At least 8 of the genes identified on chromosome 3 are associated with disease resistance such as *Glyma.03g128300* ~ glutamate synthase, *Glyma.03g128900* ~ lycopene β-cyclase, and *Glyma.03g129100* ~ pyrroline-5-carboxylate reductase, which are associated to disease resistance and resistance to oxidative stress (Antoniou et al. 2017; Kim et al. 2012; Khedr et al. 2003). We have verified that polymorphisms in the *P5CR* gene of *Brassica* *napus* are associated with resistance to *S.* *sclerotiorum* (Zhou et al. 2020).

It is well known that the R gene is the first class of disease resistance-conferring genes isolated from plants and that the leucine-rich repeat is the conserved domain of the R gene. The effects of Ras small GTPase signaling in plant disease resistance have been reported. In addition, the Dvl protein is a key protein of the classical Wnt/β-catenin signaling pathway. Not only does it mediate Wnt signal transduction from the cell membrane to intracellular domain, but also it participates in the downstream intra-nuclear formation of transcription complexes. The β-catenin signal constitutes part of an important signaling pathway that is related to the interaction between animals and diseases. It also upregulates the expression of GDP-fucose protein O-fucosyltransferase (FUT) (He et al. 2017; Wang et al. 2013). In this study, whether the identified Dvl and FUT proteins are involved in the response of soybean to *Sclerotinia* requires further confirmation because the association of Wnt signaling pathways to plant responses to pathogens has not been reported to date.

## Conclusion

It is necessary to perform haplotype association, epistatic interactive analysis, and post-GWAS to better understand the mechanism of induced disease resistance in plants. There is also a need to confirm the results using multiple models and to select QTL that have been repeatedly identified. Analysis of genes linked to the obtained association loci suggests that signals for plant anti-stress and anti-disease processes exhibit a high degree of relatedness and influence soybean’s response to *Sclerotinia* infection by a complex mechanism. The present comparative analysis was based on the use of a higher quantity of markers and a variety of analytical methods. *S.* *sclerotiorum* resistance is influenced by many genes that are involved in multiple processes, including the response to candidate genes participating in the signaling pathway of soybean’s response to *S.* *sclerotiorum* infection, and conferring resistance to WM was discovered. A total of 10 genomic regions in 7 chromosomes were detected, of which 5 tagSNPs were identified these were the peak SNPs at positions rs5589867, rs34387780, rs5734897, rs42091969, and rs37847000 on chromosomes 1, 3, 17, 20, and 13, respectively. Because the resistance of soybean to *Sclerotinia* belongs to quantitative trait inheritance, and thus meta-analysis may be appropriate to provide a deeper and more integrated knowledge of QTL and soybean signal transduction during WM infection.

## Supplementary information

Below is the link to the electronic supplementary material.

Supplementary file1 (DOC 91.5 KB)

## Data Availability

The datasets used and/or analyzed during the current study are available from the corresponding author.
