# Supplementary material for: Detection of candidate gene networks involved in resistance to Sclerotinia sclerotiorum in soybean
Source: J Appl Genet. 2021 Sep 11;63(1):1–14. doi: 10.1007/s13353-021-00654-z (PMC8755693; doi:10.1007/s13353-021-00654-z)
Supplement: Supplementary file 1 — Supplementary file1 (DOC 91.5 KB) [file 13353_2021_654_MOESM1_ESM.doc]

**Table S1** Phenotype values of the 126 soybean lines

| Name | Phenotype (mm) | Name | Phenotype (mm) | Name | Phenotype (mm) |
| --- | --- | --- | --- | --- | --- |
| Karlo RR | 28.60 | Bounty | 101.20 | Laurent | 133.70 |
| PRO 275 | 37.60 | Jutra | 101.30 | Lotus | 133.80 |
| S19-90 | 43.50 | PS46 RR | 101.80 | PS 59 | 134.20 |
| Toma | 49.20 | Kassidy | 102.00 | OAC Lakeview | 134.60 |
| PS 35 RR | 49.50 | Venus | 103.80 | Opal | 135.00 |
| Auriga | 50.80 | PR9368B07 | 104.60 | Naya | 135.50 |
| Majesta | 56.90 | AC Orford | 105.30 | S14-P6 | 136.80 |
| Prius RR | 57.70 | Pluton (SEMS01-10,53) | 106.00 | PS 36 | 136.80 |
| S04297.18 | 60.80 | Korus | 106.70 | Arva | 137.70 |
| MappleArrow | 61.00 | PR9368B25 | 107.50 | PS56 RR | 137.80 |
| PR918827 | 64.90 | 90A07 | 109.00 | 25-03R | 138.00 |
| OACProdigy | 65.80 | Delta | 109.30 | Emerson | 139.80 |
| S044280.44 | 65.80 | RD 714 | 109.80 | S04273.09 | 140.10 |
| Katrina | 72.00 | Kolia | 110.40 | PR9031LL | 140.30 |
| Savanna | 72.70 | S08-80 | 112.30 | PSX 09C10P | 140.40 |
| Damase | 74.50 | S20-F8 | 112.70 | Oria | 141.50 |
| Madison | 75.80 | PR9333B53 | 113.80 | Saska | 143.80 |
| DH 410 | 79.00 | PR9423B31 | 113.80 | 90M60 | 149.80 |
| PSX09C12P | 80.10 | Havane | 114.50 | Nova | 150.10 |
| Maple Donovan | 81.30 | LynxRR | 114.70 | S10-B7 | 150.80 |
| T0114 | 82.00 | OAC Oxford | 115.40 | SECAN 03-14 | 151.30 |
| AXN-1-55 | 82.30 | Misty (PR634197) | 116.60 | PR935413 | 152.00 |
| OACPrudence | 82.80 | Gentry | 116.60 | 90B11 | 152.70 |
| Phoenix | 83.10 | Azur | 117.90 | Bixi LL | 152.70 |
| Albinos | 83.30 | WIlliams 82 | 118.00 | Colby | 153.70 |
| OAC Champion | 84.50 | CFS0117 | 118.80 | Amasa | 153.80 |
| OAC Wallace | 84.60 | Accent | 119.50 | S05-T6 (CL 980101) | 155.00 |
| PRD 419 | 84.90 | AC Glengarry | 119.60 | Carina | 155.00 |
| Toki | 86.30 | Acora | 119.80 | Connor | 156.10 |
| Bakara | 86.70 | Victoria | 120.00 | S12-C2 | 156.30 |
| Gaillard | 87.40 | Dundas | 120.70 | 25-02R | 157.80 |
| Destiny | 87.90 | PR938626 | 121.00 | OAC Bayfield | 159.90 |
| Accord | 88.50 | DKB00-99 | 122.90 | Mario | 161.30 |
| Tundra | 90.80 | PRO 25-53 | 123.00 | PR935401 | 161.30 |
| PRO 2590R | 92.20 | S03-W4 | 123.30 | SECAN 02-13 | 163.20 |
| KG-41 | 92.40 | T2014 | 123.60 | Casino | 169.00 |
| T2042 (Cadence) | 93.20 | PR939402 | 126.50 | Supra | 169.50 |
| 91M02 (PR820226) | 93.50 | Enterprise | 126.90 | 90B43 | 173.60 |
| S04273.19 | 93.80 | Athos | 128.2 | 91M10 (PH0310) | 173.70 |
| Dares (CFS 487) | 94.00 | PS 24 | 130.20 | 90M40 | 176.10 |
| Cadence (T2042) | 97.20 | S12-A5 | 130.90 | Nattosan | 176.60 |
| S11-K2 | 97.30 | 2601R | 131.20 | PS73 | 192.40 |
